# The Use of Simple Language in Informal Forest Education as a Key to the Correct Interpretation of Sustainable Forest Management—The Experience of Poland

**DOI:** 10.3390/ijerph19095493

**Published:** 2022-05-01

**Authors:** Natalia Korcz, Emilia Janeczko, Agata Kobyłka

**Affiliations:** 1 Department of Natural Foundations of Forestry, Institute of Soil Science and Environment Management, University of Life Sciences in Lublin, Akademicka 13, 20-950 Lublin, Poland; 2 Department of Forest Utilization, Institute of Forest Sciences, University of Life Sciences in Warsaw, Nowoursynowska 159, 02-776 Warsaw, Poland; emilia_janeczko@sggw.edu.pl; 3 Department of Tourism and Recreation, University of Life Sciences in Lublin, Akademicka 13, 20-950 Lublin, Poland; agata.kobylka@up.lublin.pl

**Keywords:** educational board, forestry, text accessibility, educational infrastructure, fog index, promovolt

## Abstract

In view of the increasing conflict between society and forest management and a significant increase in the social functions of the forest, informal forest education is becoming increasingly important. In Poland, it is carried out mainly based on the field educational infrastructure, which consists, among other things, of forest educational paths equipped with educational boards. The paper presents the results of research on the assessment of the availability of texts presented on educational boards. The study was conducted on the basis of photographs of educational boards located on six educational paths in the forests of the Regional Directorate of State Forests in Lublin. Using the Google Lans application, the main text from each board was read and then analyzed in the Promovolt software program to determine the level of text accessibility using the Fog Index. The results were then compared with the opinion of respondents using an online survey, which indicated that most of the boards are written in language that is either simple, understandable to middle/high school students, or quite difficult but understandable to first-degree students. On the other hand, the respondents generally indicated the level of accessibility of the text to be easier because, in their opinion, it is enough to have a primary education to understand the content of the boards. This observation leads to the conclusion that in order for education to be more effective, simple language should be used, which can be understood by the less educated members of the population.

## 1. Introduction

Many studies indicate that the social functions of forests are becoming increasingly important [1,2,3,4], which is the result of numerous socio-economic processes related to an increase in leisure time, increased knowledge, and better education as well as environmental awareness, changing lifestyles, and greater concern for health [3]. At the same time, with the observed increase in the importance of the social functions of the forest, there are increasing expectations from society in terms of participation, especially active participation in matters relating to the management of nature, including forests [5,6]. Decisions on forest management must be made rationally and responsibly [7]. Many researchers, both in Poland and abroad [8,9], point out that society does not yet possess adequate knowledge of the principles of environmental functioning. Many people have an emotional approach to nature [10], and their knowledge is based on media reports [11], generating additional conflicts between decision-makers and various social groups [12,13,14]. Systematic and effective forestry education of the public is, therefore, necessary to prevent such situations. Leskinen [15] and Janse and Konijnendijk [16] emphasize that people’s participation in forestry can improve public environmental awareness and social acceptance of sustainable forest management. Forest education also affects the state of understanding of objects, processes, and laws that have always governed nature, thereby appreciating nature and human actions to protect it [17,18]. However, for this to be possible, it must be conducted in a simple way, using plain language [3,18].

### 1.1. Non-Formal Education in Poland

Informal forest education in Poland is developing thanks to the activities of national parks, but also, and most of all, thanks to institutions such as the State Forests which manage more than 80% of all forests in Poland [19]. In order to disseminate knowledge to the public, the State Forests undertake numerous initiatives in cooperation with national parks, local government organizations, non-profit organizations, and local groups with common interests [20].

According to the State Forests Education Activity Report, 1,852,129 [20] or 4.82% of the Polish population participated in forest education classes in 2019. The preferred form of classes was field meetings with a guide/educator on educational paths. The main recipients of active forms of education were children aged 7–15 years (43.57%) and adults over 19 years (34.64%) [20]. Educational trails are used both in active forms of education such as walks with an educator and passive forms such as walks in the forest without an educator/guide. The basic element of the development of such routes is educational boards. With respect to tourist–recreational forest management in Poland, there have already been attempts to inventory educational routes, paying particular attention to errors in the ergonomics of entire routes [21,22,23]. However, there is no information concerning the inventory of the educational boards themselves, although numerous authors have drawn attention to this topic [24].

### 1.2. Educational Boards Used in Informal Education

The design of educational infrastructure influences the way information is conveyed and emotions are aroused in people [25]. Effective interpretation gives visitors a greater sense of curiosity and delight. It leaves the visitor better informed and wanting to learn more [26,27]. According to Tsang et al. [28], signs and proper interpretation of the content on them can play a key role in changing visitor attitudes and behaviors. The work of Ballantyne et al. [29] and Walker and Moscardo [30] indicates that the use of educational boards can go beyond the specific tourist experience, contributing to the broader sustainability of education.

On the other hand, poorly designed boards may be a disturbing factor for people who want to commune with nature (e.g., boards that are too distinctive, not consistent with the environment) [31,32]. They may contain various types of errors, both factual and technical, related to the form or content of the board, its use or location, which may affect the quality of education and the overall perception of the institution managed by the area [24]. Seretny [33] points out that difficult texts with new words stacked on top of each other can significantly reduce the motivation level of learners. A poorly designed board, instead of stimulating the learning process and allowing the acquisition of new information, extinguishes engagement, creating resentment or a sense of failure. Educational boards in the forest can also be a factor that interferes with mental recovery, for example, during forest bathing [34].

In the scientific world, there have already been attempts to study the opinions of forest, trail, and museum users about educational boards [35,36,37,38]. There have also been attempts to analyze the power of attraction and the retention of focus of forest users [39], attempts to analyze the factors affecting these qualities in people stimulated by the educational boards as well as attempts to analyze the impact of the subject matter of the boards on public opinion [35,40], but still very little is known about the accessibility of the texts that are posted on them. One example describing this problem is the work of Janeczko et al. [41].

### 1.3. Plain Language in Educational Boards

Properly designed educational displays enable accurate interpretation—that is, a communication process designed to show the public the meanings and relationships of cultural and natural heritage through direct engagement with an object, artifact, landscape, or place [27]. Pettersson [42] suggests that the solution to the many problems of environmental interpretation is plain language. Plain Language makes it easy to communicate with people of different levels of education or age, resulting in effective communication. The global social phenomenon “Plain Language” has become known through the dissemination of a variety of official writing and news from different areas of life, which, when written in a professional language, was problematic for many people to understand, so they were “translated” using simple, short, common phrases [43,44,45,46].

The purpose of this research is to determine the accessibility of the main texts posted on educational boards, which are part of the equipment of educational trails.

In order to better explore the possibility of using educational boards for more effective forest education and improving understanding of sustainable forest management principles, the following research hypotheses were adopted:

1. the text of educational boards on educational trails is written in difficult language.
2. the correct determination of the level of accessibility of the text appearing on the educational boards requires a higher education.

## 2. Materials and Methods

### 2.1. Study Sites

**Scheme 1:**
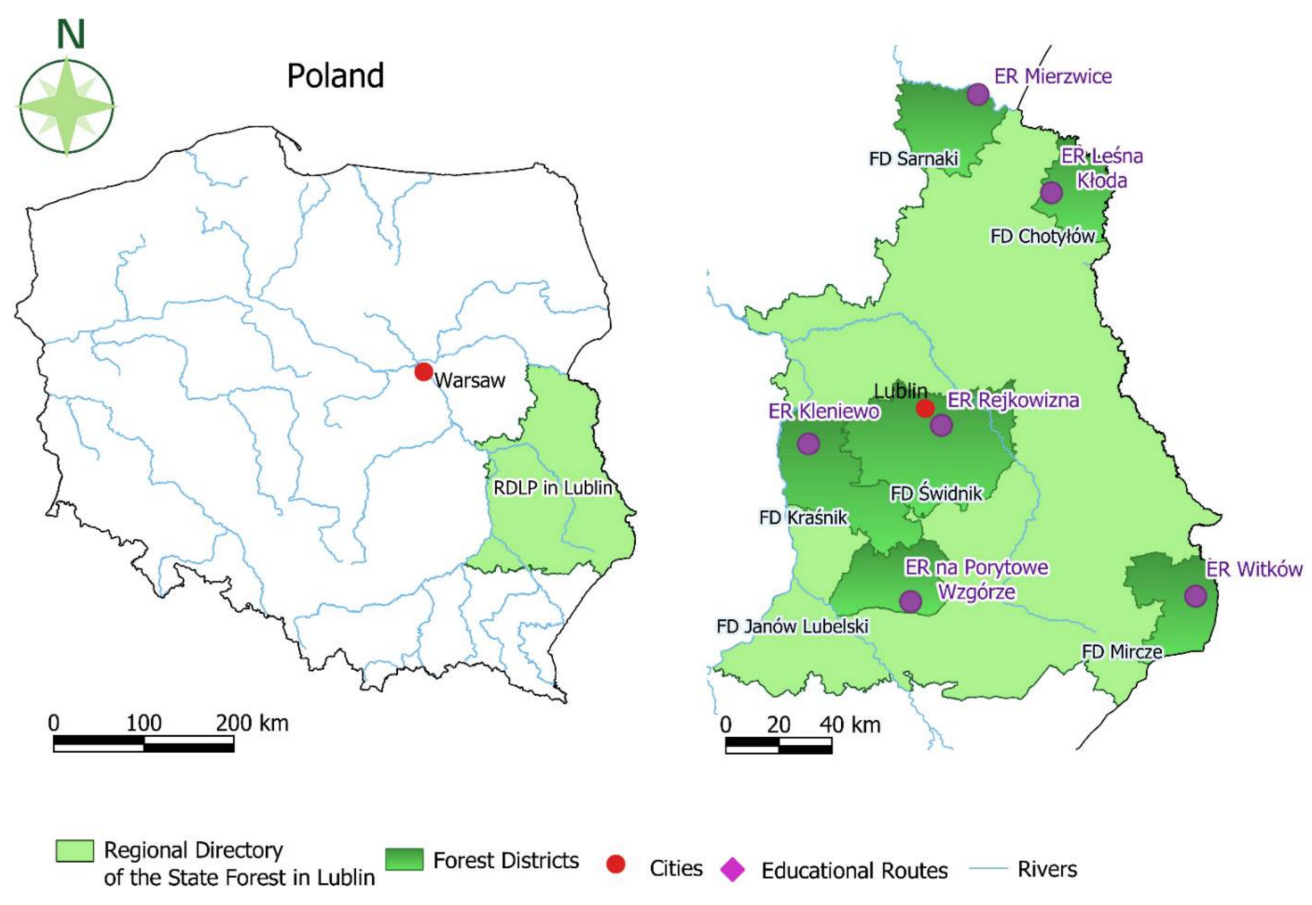
Map with location of educational routes in analyzed forest districts.

The study was carried out in the Regional Directorate of State Forests in Lublin (RDSF), which consists of 25 forest districts. The RDSF manages an area of 426 thousand ha, including 408.4 thousand ha of forest land. The forest cover in the region is 24.9%, which is one of the lowest in the country. Six educational paths located in the following Forest Districts were selected for the experiment: Chotyłów, Mircze, Sarnaki, Świdnik, Janów, and Kraśnik (Scheme 1).

These were the paths on which the largest number of educational activities were conducted in 2018 and which are the most popular among individual forest users (information received from RDSF Lublin). All routes are in the form of a loop (they start and end in the same place). Detailed analyses concerning the selection of routes for the study are described in Korcz and Janeczko [35]. Table 1 presents information on the length of routes and the number of boards.

### 2.2. Procedure

#### 2.2.1. Analysis of the Accessibility of Text and Graphics on Educational Boards

**Scheme 2:**
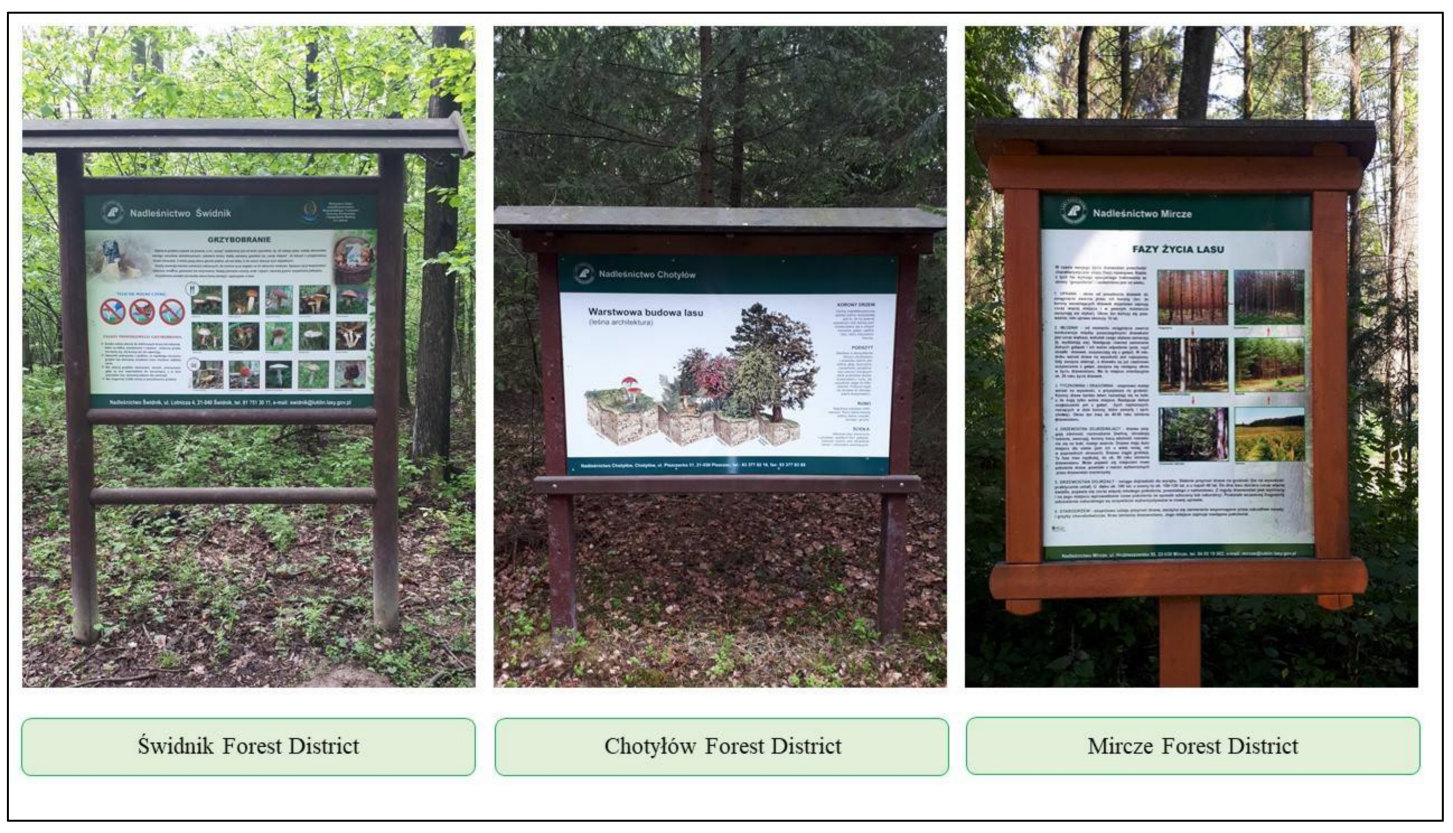
Examples of educational boards located on the analyzed educational routes.

The first stage of the study was to check the current technical condition of the educational boards on the selected trails in the field [49]. All educational boards from the 6 educational trails were in good technical condition with no signs of vandalism. Photographic documentation was also made as part of the inventory. Scheme 2 shows examples of educational boards on the routes.

Next, the text of the educational boards was analyzed in detail as part of the in-camera work. For this purpose, the main text of each board was read using the Google Lens application, then verified for linguistic correctness and analyzed using the Promovolt web application (Promovolt), available free of charge at http://www.promovolt.com [50]. Promovolt allows for the analysis of both text and images in terms of their usability, e.g., in advertising campaigns or for marketing activities [51].

This application was used to determine the number of sentences, words, multisyllabic and multi-label words, and syllables, as well as to assess the level of text comprehensibility using the Fog index (text fogginess index). The Fog index value indicates the number of years of education necessary for text comprehension [41,43,51]. The Fog index is one of the most popular indices of readability, and at the same time is adjusted to the Polish language. Its value is determined by the formula:

**Fog = 0.4 × (ASL + PDW)**

where: 

**ASL** (*Average Sentence Length*)—is the average sentence length in words,

**PDW** (*Percentage of Difficult Words*)—means the percentage of multisyllabic words (4 and more for Polish) [51].

The Fog index value can be interpreted as follows:

− 1–6: very simple text, understandable for elementary school students;
− 7–12: simple text, understandable for middle/high school students;
− 13–17: quite difficult text, understandable for first-degree students;
− 18 and above: difficult text, understandable by post-graduate students, aged over 24 years [51].

A detailed analysis of the text using the Fog index made it possible to determine the level of education needed to understand it. Based on the analyzed features, it was possible to assess the accessibility of the content of the boards. In addition, texts were also analyzed for the presence of specialized forest terminology [52], the presence of Latin names, or the presence of numerical data (units of measurement, mass, dates, etc.) [41]. The analysis was conducted in April 2019.

#### 2.2.2. An Analysis of the Accessibility of Text and on Educational Boards as Perceived by the Public

A total of 540 individuals participated in the survey. Detailed characteristics of the study participants are presented in Table 2.

For organizational reasons, it was decided to take the survey using a Google form. Surveys were distributed directly from the main profile of the authors of the paper using social media. The survey used the snowball effect [53], whereby participants were asked to forward the survey link to a minimum of two other adults (over 18 years of age), which streamlined the study and allowed it to reach as wide a range of stakeholders as possible. The survey questionnaire, in addition to the metric questions (gender, age, education level, and place of residence), included 10 questions regarding various photos on education boards (authors’ own questions and photos—see Tables 6–9 in Results). Only one answer could be selected for each question. Based on the results of the text accessibility assessment, each board was assigned to one of four categories, according to the scale proposed by Janeczko et al. [41]:

1. group 1: very simple text, understandable by elementary school students (Board 1);
2. group 2: simple text, understandable by middle/high school students (Boards 2–4);
3. group 3: rather difficult text, understandable for first-degree students (Boards 5–7);
4. group 4: difficult text, understandable for post-graduate students (Boards 8–10).

**Figure A1:**
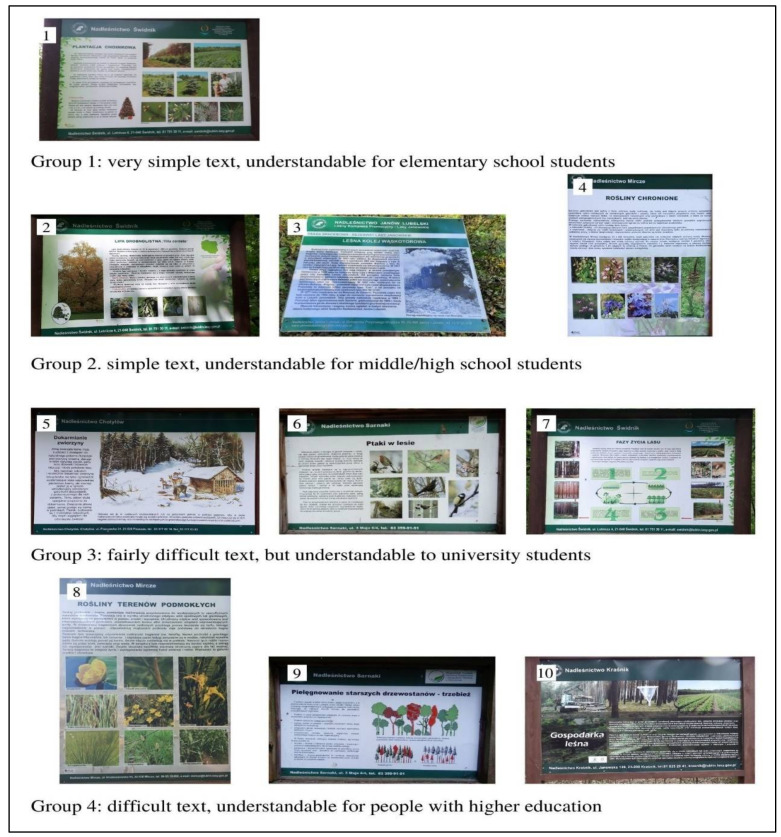
Photos of the educational boards used in the online survey.

Photos depicting the educational boards used in the questionnaire were attached to the paper as Appendix (Figure A1). On the basis of the questionnaire, it was possible to determine the respondents’ opinions on the level of accessibility of the text. In this way, it was possible to compare the results obtained with those obtained using Promovolt.

The authors give their assurance that all the procedures performed in this study were in accordance with the ethical standards of the Polish Committee on Ethics in Science and the 1964 Declaration of Helsinki, as amended.

### 2.3. Data Analysis

The individual parameters of the text on the boards were characterized using descriptive statistics (mean, standard deviation). The relationship and strength of dependence between them were also tested by calculating correlations. Pearson’s chi-2 test was used to test the significance of intergroup differences between independent samples [54].

## 3. Results

### 3.1. An Analysis of the Accessibility of Texts on the Educational Boards

The most common level of accessibility of the main text on the educational boards was simple, being understandable for middle/high school students, found on 38 boards (42.70%), and fairly difficult but understandable to first-degree students, also found on 38 boards (42.70%). The exception to this was one educational board only (1.12%), where the level of text accessibility was very simple and understandable for elementary school students. A detailed analysis of the Fog Index scores for each educational board, as determined through the use of Promovolt, is presented in Table 3.

Analysis of the results showed a negative correlation between the Fog index value and the number of sentences in the text. In other cases, the correlations were positive. As the Fog index increases, the number of sentences in the main text decreases. As the Fog index increases, the number of multisyllabic and multi-label words increases (Table 4).

As a result of the analysis, it was found that in texts rated as quite difficult but understandable for upper/high school students and difficult texts understandable for post-graduate students, forest terminology was used most often. In the case of the simple texts, understandable for middle/high school students, Latin language and numerical data were added most often to the educational boards (Table 5).

### 3.2. Analysis of the Accessibility of the Texts by the Respondents and Comparison of the Results Using Promovolt

Ratings of the accessibility of texts on educational boards were statistically significant relative to demographic factors. Table 6 shows the differences in the evaluation of the difficulty of the text on educational boards made by both genders. Statistically significant differences were obtained for board numbers 9 and 10, which were assigned to the fourth group (boards with difficult text, understandable by post-graduate students). The highest number of correct answers concerning the indication of the level of accessibility of the text concerned boards number one (group one) and two and four (group two). In both groups, the results obtained were comparable. In the case of board one, where the level of accessibility of the text is very simple and understandable for elementary school students, as many as 44.94% of females and 42.86% of males indicated the correct level of accessibility of the text. In the case of board two, where the level of accessibility of the text is simple and understandable for middle/high school students, as many as 44.30% of females gave the correct answer. In the case of board four, as many as 45.54% of males also correctly identified the level of accessibility of the text (Table 6).

Considering the educational level of the respondents, statistically significant results were obtained for the first group of boards (very simple, understandable for elementary school students), the third group (quite difficult text, understandable for first-degree students, boards 5–7), and the fourth group (difficult text, understandable for post-graduate students). Those who had a college education were significantly more likely to indicate a score adequate to that indicated by Promovolt for the third and fourth groups. The only exception is board eight (group three) (Table 7).

The age of the respondents was statistically significant for all educational boards; respondents in the 45–53 age range and those over the age of 54 most often indicated the level of accessibility of the text correctly. The exceptions are board one (group one) and board six (group three) where 55.71% of 36–44 year-olds and 14.81% of 37–35 year-olds correctly indicated the level of accessibility of the text (Table 8).

In the case of respondents’ place of residence, statistically significant results were obtained for all educational boards. Most often, people living in cities with a population of 25,000 to 100,000 correctly indicated the level of accessibility of the text (third and fourth group of educational boards). The exceptions are the boards from groups one and two. In the case of board one (group one), as many as 53.03% of respondents from cities with a population of up to 25 thousand correctly indicated the level of accessibility of the text. In the second group, in the case of boards two and three, 41.93% of respondents from cities with over 100,000 inhabitants gave the correct answer. The exception was board four (group 2), where 46.15% of respondents from a city of 25 to 100 thousand inhabitants gave a correct answer (Table 9).

## 4. Discussion

One of the key principles of creating educational materials should be using simple text, eliminating unnecessary words, and replacing scientific terms with commonly used words, or avoiding complex sentences [41,44,55]. This is especially important when creating educational materials. As Ballantyne and Hughes [56] emphasize, educational boards need to be especially effective for communication due to the presence of many random sensory stimuli that are not conducive to concentration. In addition, people’s motivation to learn, and thus to engage in interpretive media, may be greater in museums and other indoor settings compared to open-air settings [57]. Ballantyne and Uzzell [58] point out that adults and children have very different interpretations of how the natural world is perceived and understood. Therefore, the forms and methods of communication in forest education should be adjusted to maximize the effectiveness of educational boards for the general public [29,30]. Simple, easy, and brief verbal messages are better assimilated by people [59], including children [60]. According to Kim [61], it is also necessary to develop some standard elements for the design attributes of signs, which will allow more effective and reliable implementation of evaluation work related to the use of educational boards in education.

According to Taylor [62] and Munksgaard et al. [63], the Fog Index tool can be used to measure the level of simplicity of language in most types of documents as well as educational materials. The Fog Index is calculated based on, among other things, the number of long words, and statistical analysis showed a significant correlation between the scores generated by Promovolt and both the number of multisyllabic words and the average number of words in a sentence. As Pankowska and Rostkowska [52] point out, in Polish, specialized terms are in many cases four-syllable words or more, which may also explain the correlation between the number of specialized vocabulary terms and the evaluation value generated by Promovolt. Specialized forestry terminology is a factor that reduces the level of accessibility of the texts. Munksgaard et al. [63], Korcz and Janeczko [35], and Snopek [24] pointed out this aspect in their works, emphasizing the fact that educational materials must be simple and the content related to forest terminology can only be an addition or curiosity in this type of educational material.

In Poland, a worldwide social phenomenon, Plain Language, is gaining more and more supporters, whose advocates promote the idea of writing in “simple language”—the most important features of which are comprehensibility, effectiveness, and universality [44,45]. The use of plain language has also received attention in the field of medicine, creating more accessible questionnaires for sick people (a common method of health communication) [64] or in the legal context, to write more easily understandable legal documents [55,65]. The proponents of the plain language concept assume that public information does not reach the majority of society because it is transmitted in too exclusive a language—the language of a few well-educated people, too difficult to understand for the average person. This hypothesis was also partially confirmed by the results of our study.

Most of the educational boards are written in relatively simple language, understandable for middle/high school students, or 38 boards (42.70%), and fairly difficult text but understandable to first-degree students, also 38 boards (42.70%) (Table 3). Interestingly, studies conducted in the urban forests of Warsaw also give similar results [41]. The difficult level of accessibility of educational boards can be a significant problem because the largest target group of educational activities on the trails is children [20]. Among the analyzed boards, only one was adjusted to the level of education of young children. This may significantly affect the communication and education of people participating in forest education classes. However, another important problem is that the respondents incorrectly indicated the level of accessibility of the texts on the boards analyzed. Mostly, post-graduate students, over 36 years old, from larger cities, correctly indicated the level of accessibility of the text, which indicates that to interpret them correctly, one should be an educated person (Table 8 and Table 9). This is a natural phenomenon because, as Hammet and Patterson [66] and Janse and Ottitsch [67] point out, people with higher education indicate higher participation in various forms of recreation and education in forested areas and better interpret natural behaviors and natural phenomena. On the other hand, Thilden [68] emphasizes that the majority of recreational users in parks and forests did not have knowledge of the particular topics interpreted by the educational boards. Work by Burns [69] and Evans and Durant [70] indicate that the public has difficulty understanding scientific materials. This may be due to increasing literacy problems among the public [71,72].

A way to solve the problem with the level of text appearing on educational boards is, for example, to simplify educational texts. In social communication, “acting on the text”, which consists of adapting the text to strictly defined norms of accessibility (comprehensibility), has become more and more popular; a given text will gain the widest possible social range, that is, it will be understood by the so-called average citizen [67,73]. Difficult, incomprehensible professional terms are replaced by colloquial synonyms, thereby improving communication [74]. Another solution can be the replacement of written words with pictograms, figures, or pictures, which allow communication in a faster and more effective way [75]. Visual representation of content can not only evoke emotions [63], it can also portray the status of creatures, objects, or scenes occurring in nature in a realistic way, in a more attractive, objective, and appealing manner as opposed to purely textual form [76]. The application of these principles is very important today due to the increasing number of social conflicts resulting from the public’s lack of understanding of the basic principles of sustainable forest management.

## 5. Conclusions

Educational boards located on educational trails in forests of the Regional Directorate of State Forests in Lublin are not well adapted to the general public because they are written in somewhat difficult language. Most of the texts on educational boards use specialized forestry terminology, which can hinder the interpretation of the content.

Respondents’ responses in relation to the level of accessibility of the main texts on educational boards accompanying educational trails were statistically significant in relation to their demographic characteristics. Overall, respondents misinterpreted the content on the educational boards, indicating that the text on the boards is very simple and understandable for elementary school students. Respondents with higher education, over 36 years of age, and living in a city of 25–100 thousand inhabitants, most often indicated the correct level of text accessibility. The results of our study should be taken into account when creating this type of educational material to educate the public more effectively. In both theory and practice, our research can also contribute to mitigating social conflicts in forestry. Appropriate board design, using simple language, can also affect the costs associated with board preparation as well as increase other people’s interest in recreation in natural areas.

## 6. Limitations

Limitations in our work may include the relatively small group of randomly selected educational boards analyzed from a small number of routes. Nevertheless, this number was sufficient to observe some important issues that should be investigated more extensively. In the next stages of the research, the target audience should be expanded to include younger people, including children. Another important element is to investigate whether the subject matter of the educational boards is directly related to the accessibility of the texts included on those boards. Then, an attempt should be made to investigate whether the graphic design as well as the graphics themselves, contained on the educational boards, have a significant impact on the level of understanding of the content contained on the boards by the public. In addition, this type of research should be conducted in different locations (indoors and directly on educational trails) to see if the natural environment can have any influence on the interpretation of the content contained on the boards.

## Figures and Tables

**Table 1:** Technical and environmental parameters of educational pathways.

Number	Forest Districts	Route Name	Length of the Educational Route	Number of Boards	The Predominant Theme of the Boards	Predominant Function *	Forest Habitats **
1	Chotyłów	Educational route Leśna Kłoda	2 km	8	25% forest management	commercial forest	fresh mixed broadleaved forest
					25% animals in the forests		
					25% plants in forests		
					25% environmental protection		
2	Mircze	Educational route Witków	1.5 km	17	58.82% forest management	commercial forest	fresh broadleaved forest
3	Sarnaki	Educational route Mierzwice	3 km	30	23.33% forest management	commercial forest	fresh mixed coniferous forests
					23.33% plants in forests		
4	Świdnik	Educational route Rejkowizna	3.5 km	11	63% forest management	commercial forest	fresh broadleaved forest
5	Janów Lubelski	Educational route Porytowe Wzgórze	4.7 km	10	30% animals in the forests	protection forest	fresh mixed broadleaved forest
6	Kraśnik	Educational route Kleniewo	2.8 km	13	30.77% forest management	commercial forest	fresh mixed coniferous forests
					30.77% plants in forests		
Total			17.5 km	89	-	-	-

* Forest functions have been determined thanks to the Forest Data Bank website [47]; ** Forest habitat types according to the European Forest Types-European Environment Agency [48].

**Table 2:** Detailed characteristics of the respondents.

Distribution of Respondents—Demographics		n	%
Gender	Female	316	58.52
	Male	224	41.48
Age	18–26	162	30.00
	27–35	148	27.41
	36–44	140	25.93
	45–53	54	10.00
	>54 years old	36	6.67
Educational level	Primary education	40	7.41
	High school	238	44.07
	University	262	48.52
Place of residence	village	154	28.52
	city up to 25 thousand inhabitants	132	24.44
	city of 25–100 thousand inhabitants	130	24.07
	city of over 100,000 inhabitants	124	22.96

**Table 3:** Detailed analysis of primary texts on educational boards determined using Promovolt.

Variable *	µ ± SD			
	Very Simple, Understandable for Elementary School Students	Simple, Understandable for Middle/High School Students	Fairly Difficult Text, but Understandable to First Degree Students	Difficult Text, Understandable for Post-Graduate Students
	1 Board	38 Boards	38 Boards	12 Boards
Number of sentences	8.00	11.08 ± 4.97	8.26 ± 4,56	4.50 ± 2.15
Number of words	120.00	136.71 ± 63.81	157.84 ± 88.09	121.83 ± 51.15
Number of multisyllabic words	12.00	19.39 ± 9.24	26.97 ± 16.48	26.25 ± 16.15
Number of multi-label words	16.00	31.84 ± 15.50	39.16 ± 20.22	40.83 ± 19.38
Number of syllables	130.00	308.79 ± 136.36	348.92 ± 194.67	307.42 ± 126.50

µ ± SD—mean ± standard deviation; * Results from Promovolt.

**Table 4:** Correlation analysis between detailed parameters of the text.

Variable	Fog Index	Number of Sentences	Number of Words	Number of Multisyllabic Words	Number of Multi-Label Words	Number of Syllables
Fog Index	1.00					
Number of sentences	−0.43 ***	1.00				
Number of words	0.01	0.74 ***	1.00			
Number of multisyllabic words	0.29 **	0.54 ***	0.82 ***	1.00		
Number of multi-label words	0.23 *	0.58 ***	0.88 ***	0.87 ***	1.00	
Number of syllables	0.09	0.72 ***	0.96 ***	0.80 ***	0.89 ***	1.00

* Correlation is significant at the 0.05 level (2-tailed); ** Correlation is significant at the 0.01 level (2-tailed); *** Correlation is significant at the 0.001 level (2-tailed).

**Table 5:** Analysis of additional elements present in the texts of the main educational boards.

Variable	Very Simple, Understandable for Elementary School Students	Simple, Understandable for Middle/High School Students	Fairly Difficult Text, but Understandable to First Degree Students	Difficult Text, Understandable for Post-Graduate Students
	1 Board	38 Boards	38 Boards	12 Boards
	[%]			
Use of forestry terminology	100.00	57.89	68.42	66.67
Use of the Latin language	100.00	28.95	13.16	0.00
Use of numerical data	100.00	39.47	34.21	16.67

**Table 6:** Differences in the evaluation of the accessibility of the text on educational boards, taking into account the gender of respondents.

Group	Board	Gender	I Think the Text Is:				Statistics	
			Very Simple, Understandable for Elementary School Students	Simple, Understandable for Middle/High School Students	Fairly Difficult Text, but Understandable to First Degree Students	Difficult Text, Understandable for Post-Graduate Students	Chi^2 Pearson	*p* *
1	1	Female	44.94	36.71	14.56	3.80	1.915	0.590
		Male	42.86	37.50	13.39	6.25		
2	2	Female	40.51	44.30	10.76	4.43	7.172	0.067
		Male	47.32	33.04	14.29	5.36		
	3	Female	29.11	39.87	22.15	8.86	1.235	0.744
		Male	33.04	38.39	21.43	7.14		
	4	Female	31.01	39.24	20.89	8.86	2.137	0.545
		Male	27.68	45.54	18.75	8.04		
3	5	Female	66.46	24.05	5.06	4.43	5.606	0.132
		Male	66.96	19.64	9.82	3.57		
	6	Female	39.87	39.87	13.92	6.33	1.073	0.784
		Male	41.96	36.61	13.39	8.04		
	7	Female	36.08	24.68	29.11	10.13	6.083	0.108
		Male	31.25	33.93	24.11	10.71		
4	8	Female	31.01	34.81	21.52	12.66	0.261	0.967
		Male	33.04	33.93	20.54	12.50		
	9	Female	18.35	27.85	25.95	27.85	12.714	0.005 *
		Male	23.21	37.50	22.32	16.96		
	10	Female	14.56	25.95	30.38	29.11	15.418	0.001 *
		Male	25.00	31.25	20.54	23.21		

Percentage of correct assessments. * Statistically significant differences (*p* < 0.05).

**Table 7:** Differences in ratings of text accessibility on educational boards by educational level.

Group	Board	Educational Level	I Think the Text Is:				Statistics	
			Very Simple, Understandable for Elementary School Students	Simple, Understandable for Middle/High School Students	Fairly Difficult Text, but Understandable to First Degree Students	Difficult Text, Understandable for Post-Graduate Students	Chi^2 Pearson	*p* *
1	1	Primary education	35.00	35.00	20.00	10.00	21.982	0.001 *
		High school	47.06	38.66	7.56	6.72		
		University	42.75	35.88	19.08	2.29		
2	2	Primary education	30.00	40.00	20.00	10.00	10.797	0.095
		High school	43.70	41.18	9.24	5.88		
		University	45.04	28.17	13.74	3.05		
	3	Primary education	25.00	35.00	20.00	20.00	10.881	0.092
		High school	31.09	37.82	21.85	9.24		
		University	31.30	41.22	22.14	5.34		
	4	Primary education	25.00	40.00	20.00	15.00	5.533	0.477
		High school	31.93	42.86	16.81	8.40		
		University	28.24	41.22	22.90	7.63		
3	5	Primary education	50.00	30.00	5.00	15.00	18.863	0.004 *
		High school	66.39	21.01	9.24	3.36		
		University	69.47	22.14	5.34	3.05		
	6	Primary education	30.00	55.00	0.00	15.00	17.415	0.008 *
		High school	44.54	34.45	13.45	7.56		
		University	38.93	39.69	16.03	5.34		
	7	Primary education	25.00	20.00	30.00	25.00	28.352	0.000 *
		High school	36.97	32.77	18.49	11.76		
		University	32.82	25.95	34.35	6.87		
4	8	Primary education	5.00	50.00	20.00	25.00	25.808	0.000 *
		High school	36.13	36.13	15.97	11.76		
		University	32.06	30.53	25.95	11.45		
	9	Primary education	15.00	25.00	40.00	20.00	41.483	0.000 *
		High school	28.57	37.82	18.49	15.13		
		University	13.74	27.48	27.48	31.30		
	10	Primary education	5.00	25.00	50.00	20.00	45.426	0.000 *
		High school	24.37	36.13	21.85	17.65		
		University	16.03	21.37	26.72	35.88		

Percentage of correct assessments. * Statistically significant differences (*p* < 0.05).

**Table 8:** Differences in ratings of text accessibility on educational boards by age group.

Group	Board	Age	I Think the Text Is:				Statistics	
			Very Simple, Understandable for Elementary School Students	Simple, Understandable for Middle/High School Students	Fairly Difficult Text, but Understandable to First Degree Students	Difficult Text, Understandable for Post-Graduate Students	Chi^2 Pearson	*p* *
1	1	18–26	44.44	41.98	8.64	4.94	71.160	0.000 *
		27–35	48.65	35.14	16.22	0.00		
		36–44	55.71	28.57	11.43	4.29		
		45–53	25.93	33.33	22.22	18.52		
		>54 years-old	5.56	61.11	27.78	5.56		
2	2	18–26	39.51	43.21	12.35	4.94	75.134	0.000 *
		27–35	45.95	44.59	8.11	1.35		
		36–44	58.57	27.14	12.86	1.43		
		45–53	25.93	33.33	18.52	22.22		
		>54 years-old	16.67	61.11	16.67	5.56		
	3	18–26	27.16	37.04	25.93	9.88	43.413	0.000 *
		27–35	32.43	48.65	14.86	4.05		
		36–44	42.86	30.00	21.43	5.71		
		45–53	22.22	33.33	25.93	18.52		
		>54 years-old	5.56	55.56	27.78	11.11		
	4	18–26	27.16	41.98	20.99	9.88	37.473	0.000 *
		27–35	32.43	45.95	18.92	2.70		
		36–44	38.57	37.14	17.14	7.14		
		45–53	22.22	37.04	18.52	22.22		
		>54 years-old	5.56	50.00	33.33	11.11		
3	5	18–26	62.96	25.93	7.41	3.70	40.149	0.000 *
		27–35	66.22	27.03	6.76	0.00		
		36–44	77.14	12.86	4.29	5.71		
		45–53	55.56	18.52	11.11	14.81		
		>54 years-old	61.11	27.78	11.11	0.00		
	6	18–26	41.98	40.74	12.35	4.94	56.628	0.000 *
		27–35	40.54	44.59	14.86	0.00		
		36–44	48.57	28.57	14.29	8.57		
		45–53	29.63	29.63	14.81	25.93		
		>54 years-old	22.22	55.56	11.11	11.11		
	7	18–26	32.10	30.86	23.46	13.58	48.941	0.000 *
		27–35	31.08	40.54	25.68	2.70		
		36–44	44.29	18.57	28.57	8.57		
		45–53	29.63	14.81	29.63	25.93		
		>54 years-old	22.22	27.78	38.89	11.11		
4	8	18–26	35.80	37.04	14.81	12.35	48.830	0.000 *
		27–35	29.73	41.89	20.27	8.11		
		36–44	40.00	22.86	24.29	12.86		
		45–53	25.93	29.63	18.52	25.93		
		>54 years-old	0.00	44.44	44.44	11.11		
	9	18–26	23.46	38.27	24.69	13.58	53.970	0.000 *
		27–35	14.86	40.54	22.97	21.62		
		36–44	24.29	22.86	17.14	35.71		
		45–53	25.93	14.81	29.63	29.63		
		>54 years-old	5.56	27.78	50.00	16.67		
	10	18–26	18.52	39.51	24.69	17.28	81.865	0.000 *
		27–35	17.57	33.78	24.32	24.32		
		36–44	25.71	15.71	22.86	35.71		
		45–53	18.52	22.22	14.81	44.44		
		>54 years-old	0.00	11.11	72.22	16.67		

Percentage of correct assessments. * Statistically significant differences (*p* < 0.05).

**Table 9:** Differences in ratings of text accessibility on educational boards by place of residence.

Group	Board	Place of Residence	I Think the Text Is:				Statistics	
			Very Simple, Understandable for Elementary School Students	Simple, Understandable for Middle/High School Students	Fairly Difficult Text but Understandable to First-Degree Students	Difficult Text, Understandable for Post-Graduate Students	Chi^2 Pearson	*p* *
1	1	village	46.75	37.66	11.69	3.90	24.703	0.003 *
		city up to 25 thousand inhabitants	53.03	33.33	9.09	4.55		
		city of 25–100 thousand inhabitants	41.54	35.38	13.85	9.23		
		city of over 100,000 inhabitants	33.87	41.94	22.58	1.61		
2	2	village	41.56	44.16	9.09	5.19	129.50	0.021 *
		city up to 25 thousand inhabitants	56.06	30.30	10.61	3.03		
		city of 25–100 thousand inhabitants	33.85	41.54	16.92	7.69		
		city of over 100,000 inhabitants	41.94	41.94	12.90	3.23		
	3	village	31.17	42.86	18.18	7.79	24.604	0.003 *
		city up to 25 thousand inhabitants	43.94	31.82	16.67	7.58		
		city of 25–100 thousand inhabitants	21.54	40.00	26.15	12.31		
		city of over 100,000 inhabitants	25.81	41.94	27.42	4.84		
	4	village	33.77	41.56	14.29	10.39	35.189	0.000 *
		city up to 25 thousand inhabitants	42.42	36.36	16.67	4.55		
		city of 25–100 thousand inhabitants	18.46	46.15	21.54	13.85		
		city of over 100,000 inhabitants	22.58	43.55	29.03	4.84		
3	5	village	62.34	31.17	2.60	3.90	24.413	0.004 *
		city up to 25 thousand inhabitants	72.73	16.67	9.09	1.52		
		city of 25–100 thousand inhabitants	61.54	20.00	10.77	7.69		
		city of over 100,000 inhabitants	70.97	19.35	6.45	3.23		
	6	village	44.16	40.26	11.69	3.90	24.540	0.004 *
		city up to 25 thousand inhabitants	46.97	36.36	9.09	7.58		
		city of 25–100 thousand inhabitants	35.38	30.77	21.54	12.31		
		city of over 100,000 inhabitants	35.48	46.77	12.90	4.84		
	7	village	35.06	32.47	24.68	7.79	31.763	0.000 *
		city up to 25 thousand inhabitants	46.97	24.24	19.70	9.09		
		city of 25–100 thousand inhabitants	18.46	32.31	32.31	16.92		
		city of over 100,000 inhabitants	35.48	24.19	32.48	8.06		
	8	village	42.86	29.87	15.58	11.69	27.883	0.001 *
		city up to 25 thousand inhabitants	34.85	34.85	19.70	10.61		
		city of 25–100 thousand inhabitants	18.46	36.92	24.62	20.00		
		city of over 100,000 inhabitants	29.03	37.10	25.81	8.06		
	9	village	35.06	29.87	16.88	18.18	44.204	0.000 *
		city up to 25 thousand inhabitants	21.21	27.27	24.24	27.27		
		city of 25–100 thousand inhabitants	9.23	33.85	26.15	30.77		
		city of over 100,000 inhabitants	12.90	37.10	32.26	17.74		
	10	village	31.17	28.57	20.78	19.48	35.141	0.000 *
		city up to 25 thousand inhabitants	18.18	31.82	21.21	28.79		
		city of 25–100 thousand inhabitants	10.77	24.62	29.23	35.38		
		city of over 100,000 inhabitants	12.90	27.42	35.48	24.19		

Percentage of correct assessments. * Statistically significant differences (*p* < 0.05).

## Data Availability

Not available.
